# The global inhomogeneity index assessed by electrical impedance tomography overestimates PEEP requirement in patients with ARDS: an observational study

**DOI:** 10.1186/s12871-022-01801-7

**Published:** 2022-08-15

**Authors:** Serge J. H. Heines, Sebastiaan A. M. de Jongh, Ulrich Strauch, Iwan C. C. van der Horst, Marcel C. G. van de Poll, Dennis C. J. J. Bergmans

**Affiliations:** 1 Department of Intensive Care Medicine, Maastricht University Medical Centre+, P. Debyelaan 25, P.O. Box 5800, 6202 AZ Maastricht, The Netherlands; 2 Cardiovascular Research Institute Maastricht (CARIM), Maastricht University, Maastricht, The Netherlands; 3 Department of Surgery, Maastricht University Medical Centre+, P. Debyelaan 25, 6229HX Maastricht, the Netherlands; 4 School of Nutrition and Translational Research in Metabolism (NUTRIM), Maastricht University, Maastricht, the Netherlands

**Keywords:** Electrical impedance tomography, Global inhomogeneity index, Positive end-expiratory pressure, Mechanical ventilation, Respiratory system compliance

## Abstract

**Background:**

Electrical impedance tomography (EIT) visualises alveolar overdistension and alveolar collapse and enables optimisation of ventilator settings by using the best balance between alveolar overdistension and collapse (ODCL). Besides, the global inhomogeneity index (GI), measured by EIT, may also be of added value in determining PEEP. Optimal PEEP is often determined based on the best dynamic compliance without EIT at the bedside. This study aimed to assess the effect of a PEEP trial on ODCL, GI and dynamic compliance in patients with and without ARDS. Secondly, PEEP levels from “optimal PEEP” approaches by ODCL, GI and dynamic compliance are compared.

**Methods:**

In 2015–2016, we included patients with ARDS using postoperative cardiothoracic surgery patients as a reference group. A PEEP trial was performed with four consecutive incremental followed by four decremental PEEP steps of 2 cmH_2_O. Primary outcomes at each step were GI, ODCL and best dynamic compliance. In addition, the agreement between ODCL, GI, and dynamic compliance was determined for the individual patient.

**Results:**

Twenty-eight ARDS and 17 postoperative cardiothoracic surgery patients were included. The mean optimal PEEP, according to best compliance, was 10.3 (±2.9) cmH_2_O in ARDS compared to 9.8 (±2.5) cmH_2_O in cardiothoracic surgery patients. Optimal PEEP according to ODCL was 10.9 (±2.5) in ARDS and 9.6 (±1.6) in cardiothoracic surgery patients. Optimal PEEP according to GI was 17.1 (±3.9) in ARDS compared to 14.2 (±3.4) in cardiothoracic surgery patients.

**Conclusions:**

Currently, no golden standard to titrate PEEP is available. We showed that when using the GI, PEEP requirements are higher compared to ODCL and best dynamic compliance during a PEEP trial in patients with and without ARDS.

**Supplementary Information:**

The online version contains supplementary material available at 10.1186/s12871-022-01801-7.

## Introduction

The application of positive end-expiratory pressure (PEEP) is an established cornerstone in the ventilation of patients with acute respiratory failure and acute respiratory distress syndrome (ARDS) [1]. PEEP prevents alveolar collapse and improves pulmonary compliance and oxygenation [2, 3]. The level of PEEP is usually titrated upon oxygen demand according to protocols [4]. However, these protocols do not consider the individual patient characteristics that determine alveolar recruitment and recruitability, such as body mass index (BMI), lung elastance and pulmonary oedema. The physiological and biodynamic parameters obtained by the mechanical ventilator can assess individual PEEP requirements. Dynamic respiratory system compliance (Cdyn) or static compliance measured during a decremental PEEP trial is often used [5–7]. Physiologic and biodynamic parameters can also guide tidal volume settings in individuals and prevent overdistension due to excessive tidal volume [8]. A fixed tidal volume of 6 ml kg^− 1^ scaled to predicted body weight is generally safe. However, overdistension is observed in patients with ARDS, mainly if high airway driving pressures are required [9]. Consequently, a more individualised approach to determine optimal PEEP and tidal volume setting is desired to individualise the respiratory support of patients with ARDS. Electrical impedance tomography (EIT) is a non-invasive and non-radioactive bedside monitoring tool that continuously quantifies regional impedance changes. Converting this data results in dynamic images with a high temporal resolution, allowing the operator to track the response of the lung to any change in ventilator settings on a breath-by-breath basis [10, 11]. Regional overdistension (OD) and alveolar collapse (CL) can be quantified [12]. The best balance between these two mechanical EIT outcomes (i.e., ODCL) can then be used to determine the most optimal PEEP level after a PEEP trial.

Another EIT parameter is the global inhomogeneity index (GI) which quantifies the homogeneity of the tidal volume distribution. In addition, sparse evidence is available for interindividual comparison [13] and PEEP titration to optimise alveolar homogeneity [14, 15].

This study aimed to assess the effect of an incremental-decremental PEEP trial on ODCL, GI and Cdyn in patients with ARDS compared to patients without ARDS. Furthermore, to compare “optimal PEEP” based upon different EIT parameters with “optimal PEEP” based upon Cdyn.

## Methods

### Study population

Between January 2015 and August 2016, EIT was performed in mechanically ventilated ARDS patients and patients who underwent cardiothoracic surgery (CTS) as healthy pulmonary controls. ARDS was defined according to the Berlin definition [16]. All patients were admitted to our 33-bed mixed intensive care unit of a university medical centre. All patients were invasively ventilated in a pressure-controlled, time-cycled mode at a fixed driving pressure (plateau pressure minus PEEP) using an Evita-4 or Evita-XL ventilator (Dräger Medical GmbH, Lübeck, Germany). Tidal volumes between 6- and 8-ml kg^− 1^ predicted body weight were pursued. No strict institutional guidelines on PEEP setting were in use, and PEEP was set at the treating physician’s discretion, using a lung-protective strategy. Post CTS patients were usually ventilated with a PEEP of 8 cmH_2_O. Patients with the known pulmonary disease were excluded from the control group. Data are analysed retrospectively.

### Electrical impedance tomography

EIT studies were performed with an EIT-dedicated belt containing 16 electrodes placed around the patient’s chest at the fourth or fifth intercostal space at the parasternal line, and connected to an EIT monitor (Pulmovista® 500, Dräger Medical GmbH, Lübeck, Germany) with a frame rate of 20 Hz, as described previously [17]. A Low-pass filter was set applied during the EIT measurement at a cut-off frequency of 50 min^− 1^ to exclude cardiac interference as much as possible. To improve the contact quality between the electrode and skin, measurements were started 10 minutes after applying the belt. Then, the EIT monitor was connected to the mechanical ventilator to start importing the ventilator data into the EIT monitor. We applied a stepwise increase of PEEP during the EIT measurements using steps of 2 cmH_2_O, first incremental and then decremental, until we saw the loss of end-expiratory lung impedance (EELI), reflecting derecruitment. Afterwards, an offline analysis was performed to evaluate the functional EIT images. Using the EITdiag software (EITdiag, Dräger Medical GmbH, Lübeck, Germany), tidal recruitment, end-inspiratory CL and OD could be assessed from raw EIT data at each PEEP level [17]. Our PEEP trial analysis focused on the first four consecutive incremental steps and the same PEEP levels during the decremental phase. The level of PEEP from mutual patients may differ, but the incremental and decremental PEEP steps always consist of the same four PEEP levels in both the incremental and decremental phases. Although individual patients may have had more steps, we compared the data with the same number of PEEP steps for statistical analysis.

### Calculated parameters

The calculated parameters OD, CL, GI and ODCL, were calculated automatically offline using the EITdiag software at each PEEP level. Dynamic respiratory system compliance was automatically transferred from the ventilator to the EIT monitoring device.

#### Global inhomogeneity index (GI)

The GI is a parameter quantifying the homogeneity of tidal volume distribution [13]. The image matrix in EIT consists of 32 × 32 pixels. Global inhomogeneity was calculated as the sum of the absolute differences between the median value of tidal variation and every single pixel value, divided by the sum of all impedance values, to normalise the calculated values. Only pixels within the ventilated area were used for the calculation of GI. A pixel whose regional impedance change exceeds 15% of the maximum impedance change in at least one PEEP level is considered ventilated during the PEEP trial. The smaller the GI, the more homogeneous the tidal volume is distributed within the ventilated area. A GI of zero represents a perfectly homogeneous distribution of ventilation. The global inhomogeneity index was calculated according to eq. 1.

1 $$\mathrm{GI}\ \left(\%\right)=\frac{\sum_{\mathrm{x},\mathrm{y}\in \mathrm{lung}}\left|{\mathrm{DI}}_{\mathrm{x}\mathrm{y}}\right.-\mathrm{Median}\left.\left({\mathrm{DI}}_{\mathrm{lung}}\right)\right|}{\sum_{\mathrm{x},\mathrm{y}\in \mathrm{lung}}{\mathrm{DI}}_{\mathrm{x}\mathrm{y}}}x\ 100$$

(DI_xy_ represents the impedance change value of pixel (x,y) in the identified lung area, whereas DI_lung_ represents matrix of all the pixels impedance change values within the ventilated lung area)

#### Overdistension (OD) and collapse (CL)

Overdistension and CL quantify the amount of overdistension and collapse in the lung by calculating pixel compliance at every PEEP step. Cumulated OD and CL per PEEP level were determined as the percentage change in compliance for each pixel in relation to its “best compliance” according to eqs. 2 and 3 [12].

2 $$\mathrm{Cumulated}\ \mathrm{overdistension}\ \left(\%\right)=\frac{\sum_{\mathrm{Pixel}=1}^{1024}\left({\mathrm{Overdistension}}_{\mathrm{pixel}}\left(\%\right)\times {\mathrm{Best}\ \mathrm{compliance}}_{\mathrm{pixel}}\right)}{\sum_{\mathrm{Pixel}=1}^{1024}\left({\mathrm{Best}\ \mathrm{compliance}}_{\mathrm{pixel}}\right)}$$

3 $$\mathrm{Cumulated}\ \mathrm{collapse}\ \left(\%\right)=\frac{\sum_{\mathrm{Pixel}=1}^{1024}\left({\mathrm{Collapse}}_{\mathrm{pixel}}\left(\%\right)\times {\mathrm{Best}\ \mathrm{compliance}}_{\mathrm{pixel}}\right)}{\sum_{\mathrm{Pixel}=1}^{1024}\left({\mathrm{Best}\ \mathrm{compliance}}_{\mathrm{pixel}}\right)}$$

Subsequently, the absolute difference between OD and CL (ODCL) is calculated per PEEP level. With regard to ODCL, the “optimal” PEEP level is considered the PEEP level corresponding to the lowest ODCL.

By continuously monitoring changes in Cdyn during a decremental PEEP trial, it is possible to identify the occurrence of lung collapse [5]. When Cdyn starts to decrease, it is assumed that derecruitment starts [18]. Therefore, the PEEP level with the highest Cdyn represents the optimal PEEP for that patient.

### Optimal PEEP settings

Individual optimal PEEP levels were defined according to three different biodynamic parameters: ODCL, GI and Cdyn. According to these different parameters, PEEP would theoretically be optimal when either ODCL was as close as possible closest to zero, GI was lowest, or Cdyn was highest. The increase in PEEP during the PEEP trial was stopped when no ongoing recruitment and/or significant OD occurred, despite that GI may still be decreasing. In our analysis, the PEEP with the lowest GI value during the decremental PEEP trial was considered optimal PEEP. Since, in most individual cases, no plateau for Cdyn was observed, optimal PEEP according to Cdyn could not formally be calculated in all patients. In cases no plateau was observed in Cdyn, the PEEP at the end of the decremental PEEP trial was used as the “highest estimate of optimal PEEP”. Optimal PEEP based on ODCL, GI and Cdyn will be compared for patients with ARDS and control patients.

### Statistics

Data are presented as a number (percentage) for categorical variables and mean ± standard deviation (SD) for continuous variables. Characteristics between ARDS and control patients were compared with an independent sample t-test; differences between optimal PEEP based on ODCL, GI and Cdyn were compared with paired sample t-test. Changes in ODCL, GI and changes in Cdyn during the incremental and decremental PEEP trial, as well as the effect of ARDS vs. healthy lungs on these changes, were tested using repeated measures two-way ANOVA. Individual pair-wise differences between the PEEP with ODCL closest to zero, the lowest GI and highest Cdyn were analysed by Pearson’s correlation coefficients. The agreement was assessed using Bland-Altman plots by plotting the mean of the two measurements against their difference and 95% limits of agreement (= mean difference ± 1.96 x SD of the difference). Statistical analyses were performed using SPSS version 23 software (IBM SPSS, Armonk, NY, USA). GraphPad Prism version 5.03 software (GraphPad Software, inc. La Jolla, CA, USA) and MATLAB R2013b (Mathworks, Natick, MA) generated graphs. All *p*-values < 0.05 are considered to be statistically significant.

### Ethics

Electrical impedance tomography is used as part of routine care in our department, and indications for EIT were determined based on clinical grounds. Furthermore, all procedures performed in studies were in accordance with the ethical standards of the institutional and/or national research committee and with the 1964 Helsinki Declaration and its later amendments or comparable ethical standards. Therefore, the ethics committee of the Maastricht University Medical Centre+ approved the utilisation of the collected data for scientific evaluation and individual informed consent was waived (METC 15–4-186).

## Results

A total of 28 ARDS (age 63 ± 16 years and 61% male) and 17 CTS patients (age 66 ± 14 years and 82% male) were included. Baseline demographic and clinical characteristics showed by definition a significant difference in APACHE II, PaO_2_/FiO_2_, PEEP and FiO_2_ (Table 1).

**Table 1:** Patient characteristics

	ARDS	Control	p-value
Number of patients (%)	28 (62)	17 (38)	
Sex, M/F	17/11	14/3	
APACHE II (SD)	28 (8)	17 (5)	< 0.001*
Age, years (SD)	63 (16)	66 (14)	0.527
BMI, kg/m^2^ (SD)	26 (5)	27 (5)	0.507
Tidal volume, ml/kg PBW (SD)	7.7 (2.1)	7.2 (1.1)	0.372
PaO_2_/FiO_2_, mmHg (SD)	137 (49)	339 (73)	< 0.001*
Set PEEP, mmHg (SD)	12.2 (2.6)	7.9 (0.5)	< 0.001*
FiO_2_, % (SD)	0.67 (0.19)	0.39 (0.07)	< 0.001*
Cdyn, ml/cmH_2_O (SD)	43 (20)	54 (15)	0.0499*
**Type of admission**			
Post CTS, n (%)		17 (38)	
Pneumonia, n (%)	16 (36)		
Sepsis, n (%)	9 (20)		
Other, n (%)	3 (7)		
**ARDS**			
Mild, n (%)	2 (7)		
Moderate, n (%)	19 (68)		
Severe, n (%)	7 (25)		

Data are presented as means ±SD and as the number of patients (%), where appropriate. APACHE II, Acute Physiology And Chronic Health Evaluation II; BMI, body mass index; PBW, predicted body weight; PEEP, positive end-expiratory pressure; Cdyn, dynamic respiratory system compliance; CTS, postoperative cardiothoracic surgery patients; ARDS, acute respiratory distress syndrome. (* *p <* 0.05)

### Changes in Cdyn during the PEEP trial

**Fig. 1:**
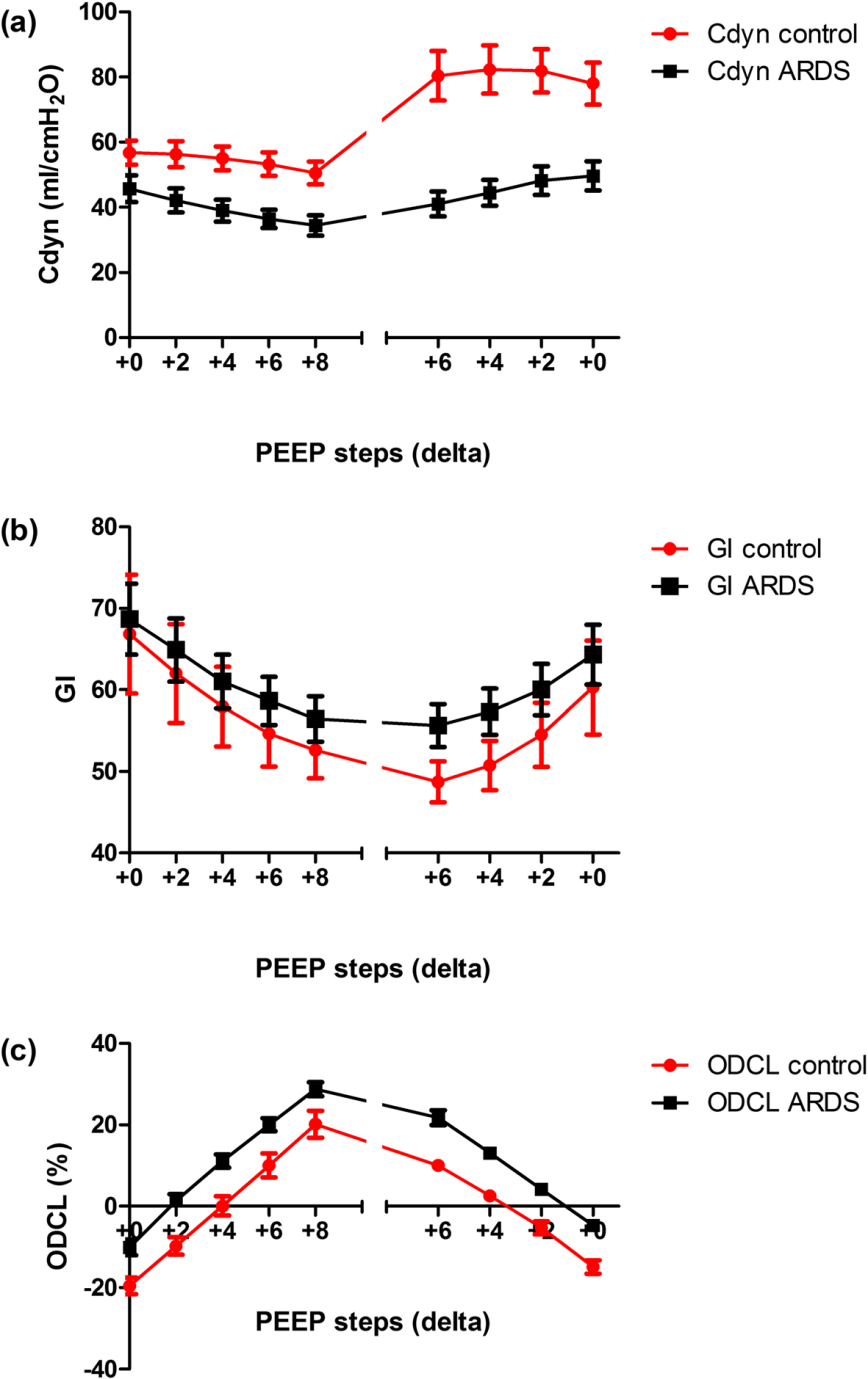
Changes in Cdyn (**a**), GI (**b**) and percentages of ODCL (**c**) in patients with ARDS (square, black) and control patients (circle, red) during an incremental and decremental PEEP trial. (*p* < 0.0001, *p* = 0.541, *p* = 0.913 respectively) ODCL, balance between alveolar overdistension and collapse; GI, global inhomogeneity index; Cdyn, dynamic respiratory system compliance

Dynamic compliance was significantly lower in ARDS patients than in controls at all PEEP levels (Fig. 1a). A plateau of Cdyn during the PEEP titration was not seen in ARDS patients compared to control patients with a plateau when PEEP was between + 2 and + 6 cmH_2_O. Mainly in ARDS, the decremental PEEP trial was stopped before such a plateau could be reached due to the occurrence of lung derecruitment evidenced by loss of end-expiratory lung impedance measured by EIT.

### Changes in ODCL during the PEEP trial

Changes in ODCL were directly related to changes in PEEP (Fig. 1c). ODCL in ARDS and control patients showed corresponding findings (*p* = 0.913). ODCL equals 0 later during the decremental PEEP trial in ARDS compared to control patients. However, PEEP in ARDS patients was on average 4 cmH_2_O higher than in controls at the start of the PEEP trial (*p <* 0.001).

### Changes in GI during the decremental PEEP trial

**Fig. 2:**
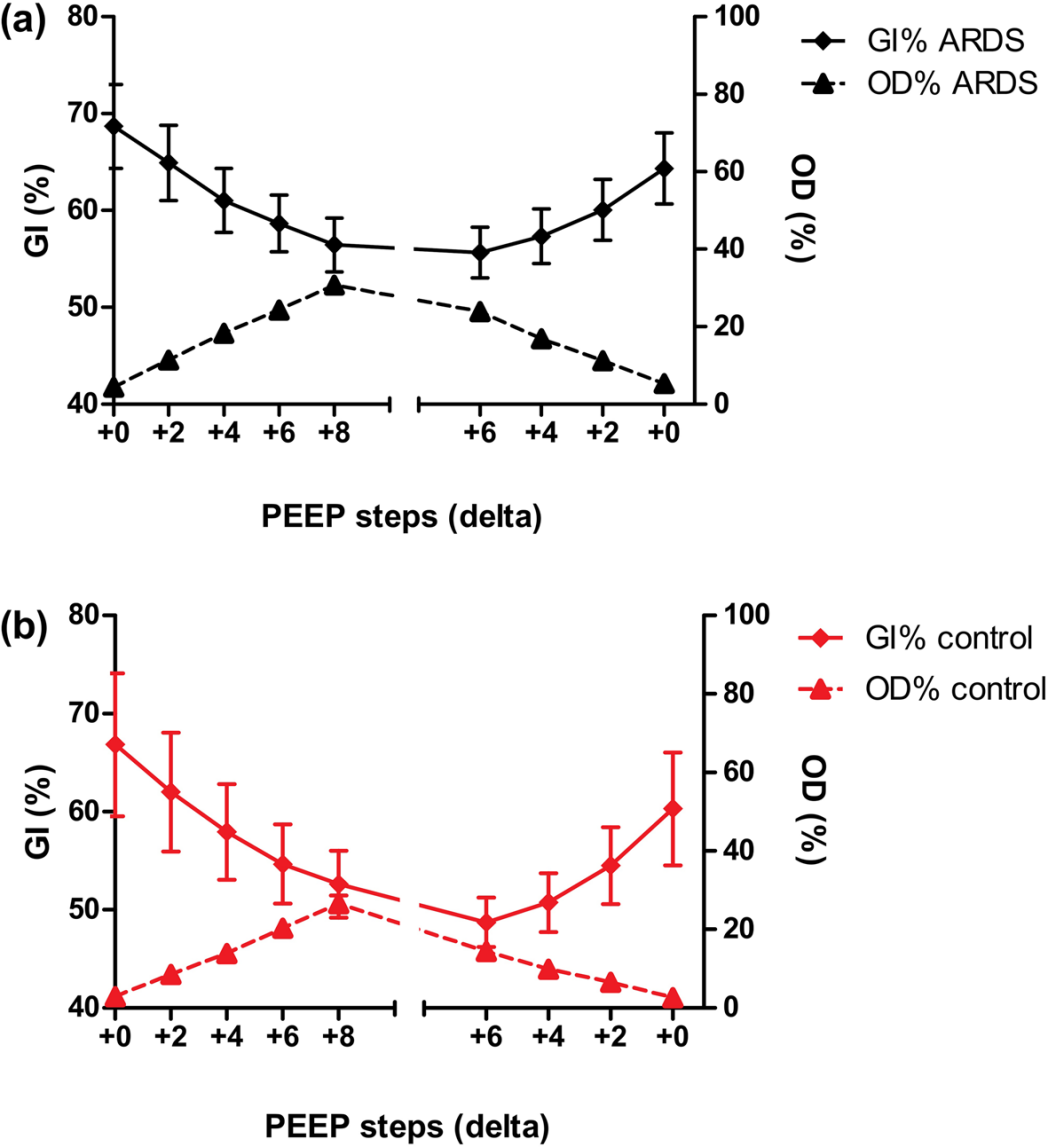
Changes in the global inhomogeneity index (GI, diamond) and the percentage of overdistension (OD, triangle) in patients with ARDS (**a**) and control (**b**) patients during an incremental and decremental PEEP trial. (*p* < 0.001, *p <* 0.001 respectively)

The GI decreased gradually during the incremental PEEP trial and increased during the decremental PEEP trial showing corresponding findings between ARDS and control patients (*p* = 0.541); no plateau level suggestive of an optimal PEEP was observed (Fig. 1b). A low GI was correlated with high OD. Both groups showed a significant interaction between GI and OD (*p <* 0.001). (Fig. 2a, b).

### Optimal PEEP

Optimal PEEP defined by ODCL, GI and Cdyn were higher in ARDS patients than in control patients. ODCL reached zero in both ARDS and control patients. On average, optimal PEEP using ODCL was equal to 10.9 cmH_2_O in ARDS patients and 9.6 cmH_2_O in control patients. GI failed to reach a plateau in all but 9 patients. The PEEP with the most homogenous ventilation distribution was 17.1 cmH_2_O in ARDS patients and 14.2 cmH_2_O in controls. The mean optimal PEEP according to the highest Cdyn was 10.3 cmH_2_O in ARDS patients and 9.8 cmH_2_O in control patients (Table 2).

**Table 2:** Optimal PEEP settings

PEEP based on	ARDS	Control	p-value
best ODCL, cmH_2_O (SD)	10.9 (2.5)	9.6 (1.6)	0.092
lowest GI, cmH_2_O (SD)	17.1 (3.9)	14.2 (3.4)	0.608
best Cdyn, cmH_2_O (SD)	10.3 (2.9)	9.8 (2.5)	0.864

Data are presented as means ± SD. ODCL, balance between alveolar overdistension and collapse; GI, global inhomogeneity index; Cdyn, dynamic respiratory system compliance; ARDS, acute respiratory distress syndrome

There was a significant difference between optimal PEEP based on ODCL and GI, as well as GI and Cdyn for both ARDS and control patients (*p* < 0.05) but not for ODCL versus Cdyn (*p* = 0.161 and *p* = 0.826, respectively). For example, PEEP based on ODCL minus PEEP with lowest GI resulted in a difference of − 6.2 cmH_2_O in patients with ARDS. Pearson’s correlation coefficients between optimal PEEP approaches are given in Table 3.

**Table 3:** Differences between optimal PEEP based on ODCL, GI and Cdyn

	ARDS	p-value	Correlation (r)	Control	p-value	Correlation (r)
**ODCL - GI**	−6.2 (3.3)	< 0.001*	0.535	−4.6 (3.8)	< 0.001*	0.030
**ODCL - Cdyn**	0.6 (2.1)	0.161	0.711	−0.1 (2.2)	0.826	0.526
**GI - Cdyn**	6.8 (3.1)	< 0.001*	0.622	4.5 (4.4)	0.001*	0.110

Data are presented as means ±SD. ODCL, balance between alveolar overdistension and collapse; GI, global inhomogeneity index; Cdyn, dynamic respiratory system compliance; ARDS, acute respiratory distress syndrome; r, Pearson’s correlation coefficient (*p < 0.05)

**Fig. 3:**
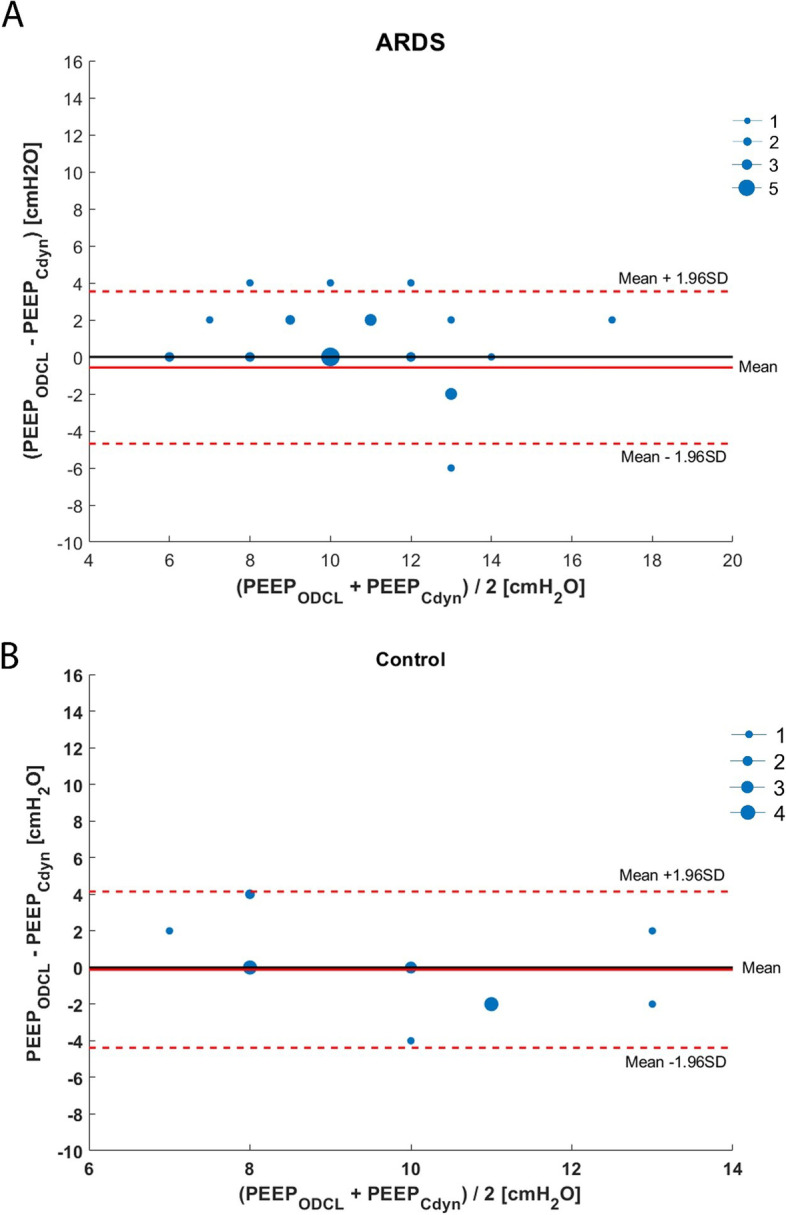
Bland-Altman plot of differences in PEEP based upon the best balance between alveolar overdistension and collapse (ODCL) and best dynamic respiratory system compliance (Cdyn) in patients with ARDS (**a**) and control patients (**b**). The dot size is an indicator for the incidence of the value. The black full line indicates no difference in PEEP level between ODCL and best Cdyn; the red full line indicates the mean value of the two measurements, and the red dashed line indicates the mean of the two measurements against their difference and 95% limits of agreement (= mean difference ± 1.96 x SD of the difference)

In 13 out of 28 (46%) ARDS patients, PEEP according to ODCL was equal to the PEEP as determined with the best Cdyn, and in 7 out of 17 (41%) control patients (Fig. 3a, b).

**Fig. 4:**
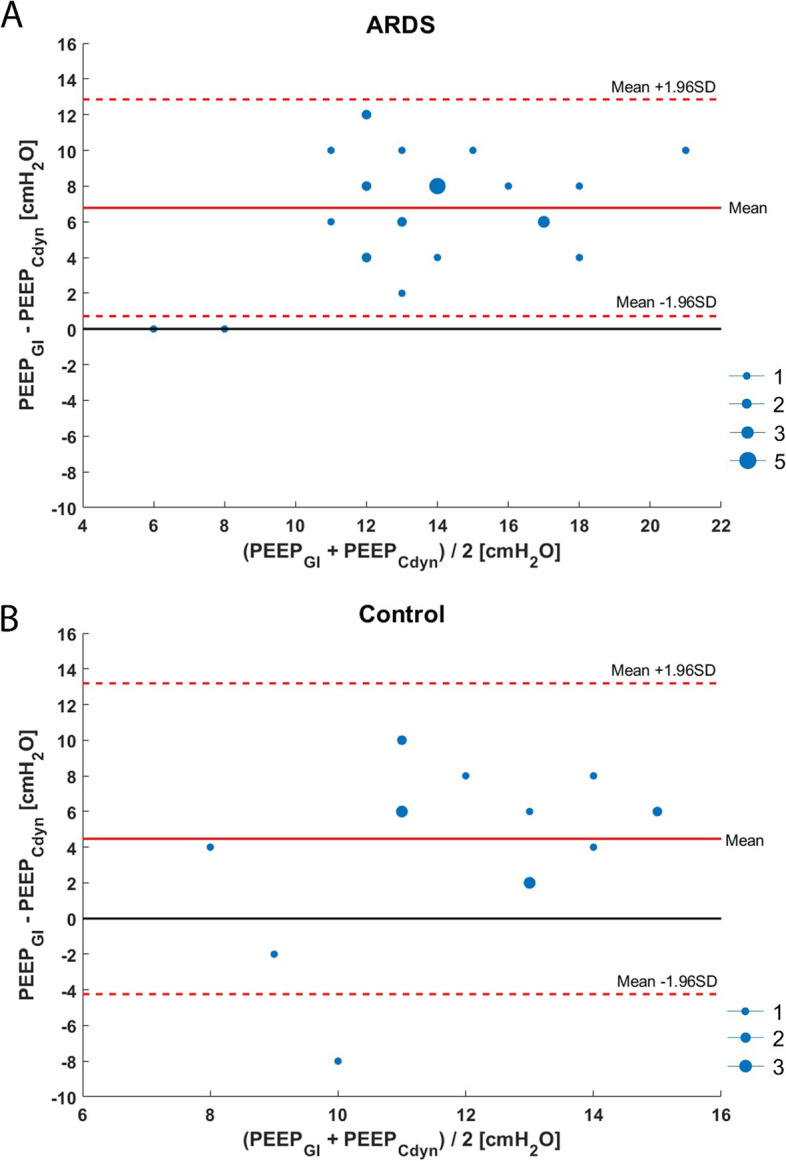
Bland-Altman plot of differences in PEEP based upon lowest global inhomogeneity index (GI) and best dynamic respiratory system compliance (Cdyn) in patients with ARDS (**a**) and control patients (**b**). The dot size is an indicator for the incidence of the value. The black full line indicates no difference in PEEP level between lowest GI and best Cdyn; the red full line indicates the mean value of the two measurements, and the red dashed line indicates the mean of the two measurements against their difference and 95% limits of agreement (=mean difference ± 1.96 x SD of the difference)

In 2 out of 28 (7%) cases, PEEP, according to the lowest GI value, was equal to the PEEP with the best Cdyn in patients with ARDS and 4 out of 17 (24%) in control patients. The agreement between PEEP based upon the most homogeneous distributed ventilation and best Cdyn is displayed in Fig. 4a, b.

**Fig. 5:**
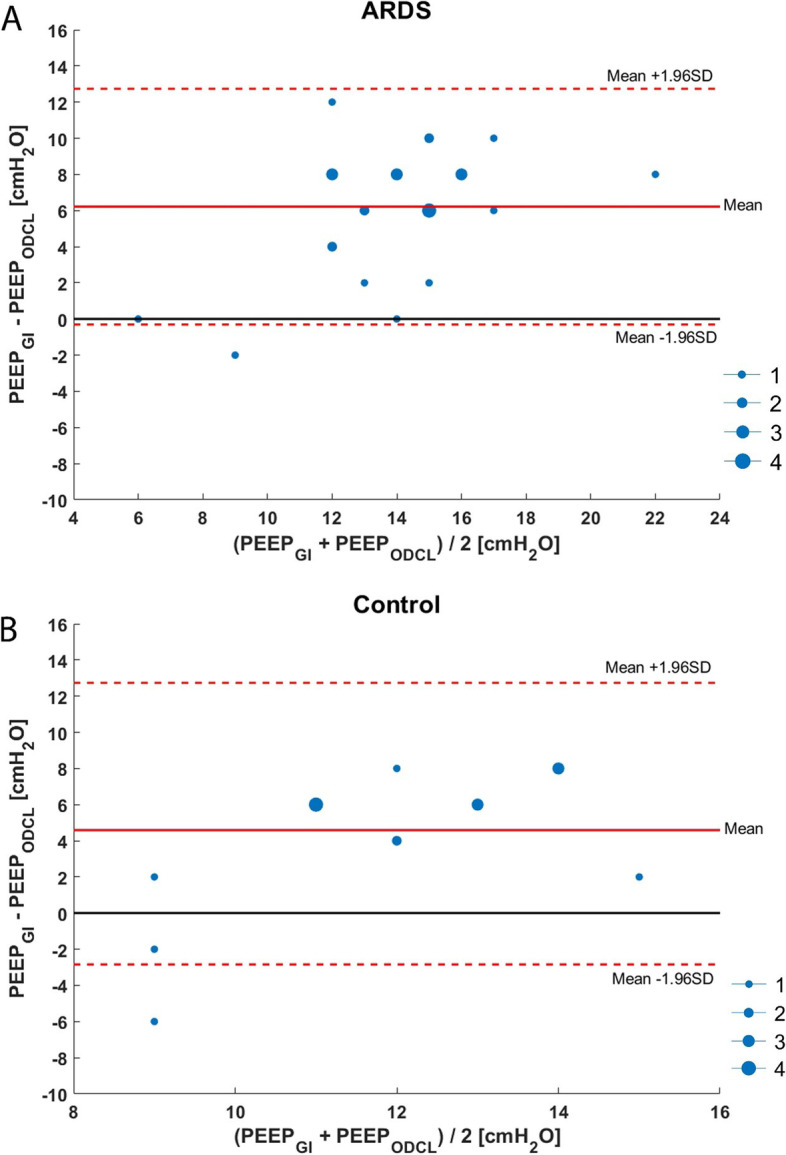
Bland-Altman plot of differences in PEEP based upon lowest global inhomogeneity index (GI) and the best balance between alveolar overdistension and collapse (ODCL) in patients with ARDS (**a**) and control patients (**b**). The dot size is an indicator for the incidence of the value. The black full line indicates no difference in PEEP level between lowest GI and best ODCL; the red full line indicates the mean value of the two measurements, and the red dashed line indicates the mean of the two measurements against their difference and 95% limits of agreement (=mean difference ± 1.96 x SD of the difference)

In 2 out of 28 (7%) cases, PEEP according to the lowest GI value was equal to the PEEP according to ODCL in patients with ARDS and 0 out of 17 (0%) cases in control patients. The agreement between PEEP based upon the most homogeneous distributed ventilation and ODCL is displayed in Fig. 5a, b.

**Fig. 6:**
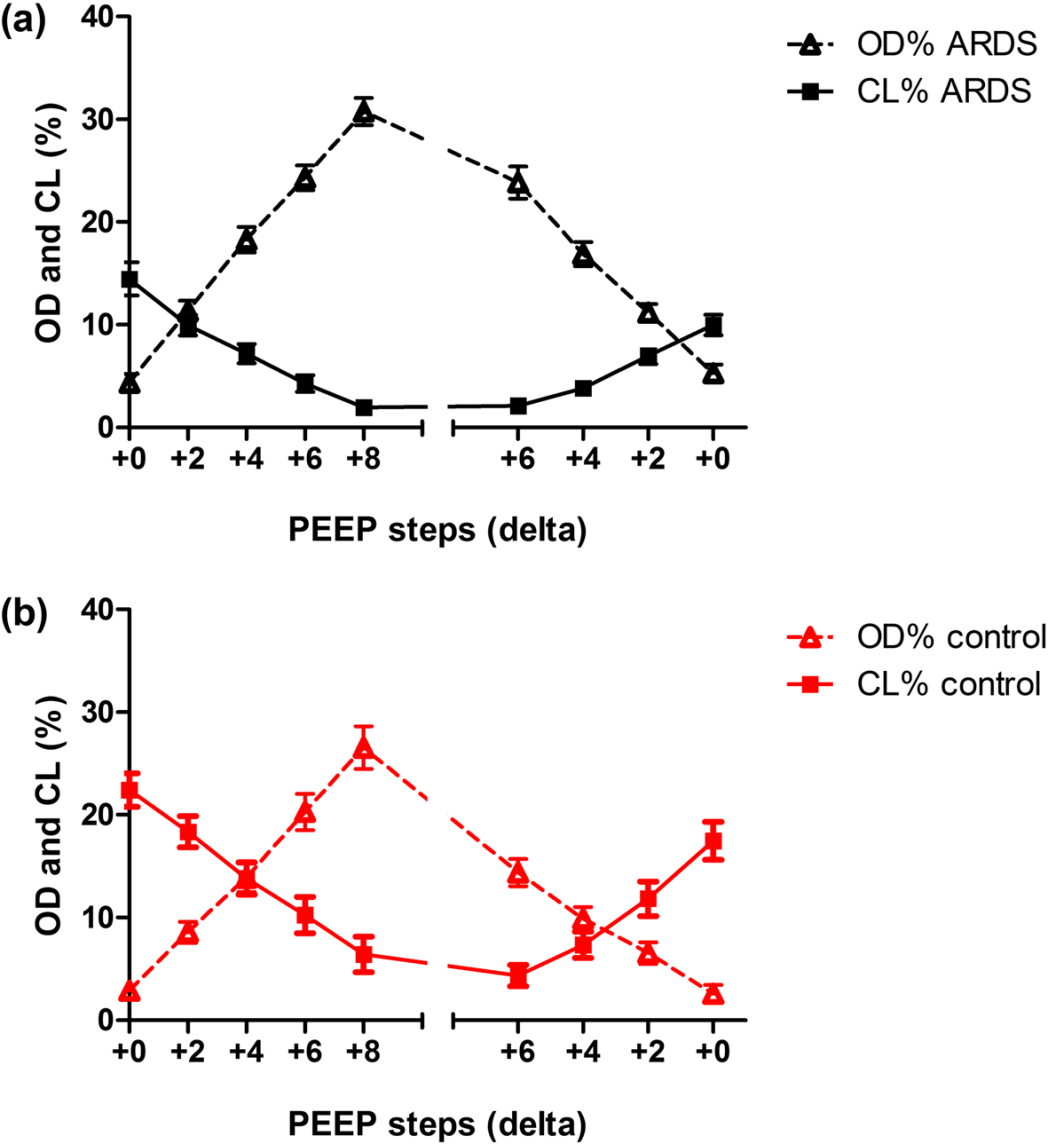
Changes in the percentage of alveolar overdistension (OD; triangle) and collapse (CL; square) during an incremental and decremental PEEP trial in patients with ARDS (**a**) and control patients (**b**). The changes in percentages are significantly different between OD and CL during the PEEP trial in both ARDS as control patients (*p <* 0.0001)

The increase in alveolar OD is more pronounced than the decrease of CL by increasing PEEP in ARDS and controls (*p* < 0.0001; Fig. 6a, b).

## Discussion

This study showed that it is feasible to use EIT to visualise regional changes in OD, CL and homogeneity of ventilation distribution during different ventilator settings in patients with ARDS and control patients in a clinical setting. These indices may help the physician decide on ventilator settings in an individual patient. Optimal PEEP based on GI showed a significant difference compared to optimal PEEP based on ODCL and best Cdyn for both ARDS and CTS patients.

No different response of ODCL and GI between patients with injured lungs (ARDS) and patients with healthy lungs during PEEP changes was observed. However, the difference in the increase of Cdyn in control patients was more pronounced. This might be explained by the fact that healthy lungs, in this case, CTS patients, are more susceptible to alveolar recruitment due to the prevalence of postoperative atelectasis [19]. Moreover, ARDS patients had a trend towards a lower Cdyn. This may explain the larger increase of OD compared to the decrease in CL in these patients. The decrease in Cdyn with increasing PEEP may indicate evident OD with the higher PEEP levels in these patients.

End expiratory impedance distribution varies widely among patients, especially in ARDS. Franchineau et al. found a broad variability in optimal PEEP in patients with severe ARDS under extracorporeal membrane oxygenation. In these patients, they found no PEEP level combining no CL and no OD, meaning that there is always a combination of a certain level of CL and OD [20]. No single optimal PEEP level exist for the whole lung [21]. This reinforces the need for personalised titration, not only of PEEP level but also tidal volume because the latter also contributes to tidal recruitment and OD [22, 23]. Therefore, EIT might be a helpful tool because it can estimate regional OD and CL percentages in individual patients. Therefore, not only PEEP but also tidal volume (driving pressure) can be adjusted according to the EIT findings.

It has been suggested that the optimal PEEP level is determined when air is most homogenously distributed in the lung [14]. Homogenisation of ventilation distribution somewhat became synonymous with a lung-protective ventilation strategy, assuming that recruited alveoli improve ventilation distribution as part of the tidal volume, thereby minimising OD as well [24, 25] We found that increasing airway pressure enhances homogenous ventilation distribution in ARDS and control patients. Nevertheless, the most homogenous ventilation resulted in the largest amount of OD in both groups. In ARDS, the increase in homogeneity with increasing airway pressure was less pronounced in severe ARDS compared to mild and moderate ARDS, suggesting that higher pressures are probably required for more pronounced homogeneous distribution of ventilation, but at the price of a higher risk of OD [26]. Using the GI solely to adjust PEEP settings in ARDS can lead to severe OD and barotrauma [27]. Solely trying to minimise inhomogeneity without limiting the upper level of PEEP may lead to severe OD and can be harmful [28]. In fact, some heterogeneity in ventilation distribution is physiologic and thus occurs in healthy subjects as well [29]. Hochhausen et al. used the combination of GI and ODCL for PEEP setting. Optimal PEEP was defined when GI had the lowest value, provided ODCL was ≤10% [30]. Combining these EIT indices may lead to a feasible and safe PEEP setting. Changes in GI at higher PEEP levels are very small. Yang et al. found that GI varies ~ 4% in healthy, spontaneously breathing volunteers [31]. Taken this into account, PEEP is still higher but considerable lower in ARDS patients in our population (12.6 cmH_2_O versus 17.1 cmH_2_O). However, correlation with ODCL and Cdyn is very weak, namely in the control group, up to moderate in patients with ARDS (supplementary information). An exactly threshold should be defined for mechanically ventilated patients without spontaneous breathing activity. Furthermore, we found that mainly in ARDS, the more homogeneously ventilation was distributed, the lower Cdyn was. This may explain the increase in OD. Maybe the GI helps identify responders and non-responders to alveolar recruitment. We hypothesise that in patients who do not respond to alveolar recruitment, GI does not change, while in responders, the GI improves due to alveolar recruitment, inducing changes in ventilation distribution [32–34]. The GI is highly correlated with lung recruitability. The percentage of recruitable lung regions decreases when the GI decreases [35, 36]. The lack of improvement in GI following a PEEP increase indicates a negative response to PEEP and warns against high PEEP levels.

To this point, there is no agreement on the gold standard method for PEEP titration. A commonly used bedside tool to determine optimal PEEP is the PEEP level with the best Cdyn during a decremental PEEP trial [5]. We found that optimal PEEP according to the best Cdyn was not in agreement with the lowest value in GI (Fig. 4). Therefore, we consider the GI solely is not suitable for optimal PEEP determination. Simultaneously, the single value Cdyn does not reflect the PEEP dependent changes in respiratory system mechanics (i.e., CL, OD and atelectrauma) [37]. Dynamic respiratory system compliance is an average over the whole tidal volume and does not give any information on the regional ventilation distribution [38]. Our results show that the application of Cdyn may lead to an underestimation of the optimal PEEP level in patients with ARDS. The optimal PEEP based on the best Cdyn in ARDS in this paper may be erroneous lower in cases no plateau for Cdyn was observed in the decremental PEEP phase. Dynamic respiratory system compliance could increase by further decreasing PEEP, which could not be assessed using our protocol. In this retrospective analysis, PEEP was set according to EIT information and not based upon best Cdyn. The advantage of EIT over Cdyn is that it can identify the level of PEEP where derecruitment begins, even if global Cdyn still increases due to some relief of OD, which can also be visualised with EIT [17]. Also, Franchineau et al. showed that PEEP set at the best Cdyn did not necessarily correspond to the PEEP level with the lowest level of CL and OD in patients with ARDS [20]. Global respiratory mechanics parameters like Cdyn for PEEP titration underestimate measures of regional ventilation distribution. Hyperinflation exists when the best respiratory system compliance is used for PEEP titration [39, 40]. A low ODCL does not exclude the presence of OD or CL, most notably if ventilation is heterogeneously distributed.

There is a strong tendency to personalise ventilator settings, particularly in conditions like ARDS where lung damage is heterogeneously, and large individual differences exist across patients [41]. However, from a physiological point of view, an individual approach to select the level of PEEP and tidal volume to the patient’s specific lung mechanics seems reasonable [42]. Furthermore, individual responses to PEEP and tidal volume should be expected in a patient with ARDS and in patients with healthy lungs [37].

## Conclusions

Individual monitoring of regional lung mechanics like ODCL, GI and Cdyn during an incremental-decremental PEEP trial, using EIT is feasible. Furthermore, the effect of different PEEP levels and tidal volumes can be reliably assessed. Our data in this study suggests selecting the PEEP level with the lowest amount of CL and decreasing airway driving pressure when large OD is observed in this specific PEEP level. Using solely Cdyn seems a good alternative to determine optimal PEEP if EIT is unavailable. We consider GI solely not suitable for determining optimal PEEP. It may lead to severe OD and is potentially harmful, while changes in GI during the PEEP trial can be used to notify alveolar recruitability. Agreement on which indices or combinations of EIT derived calculations should be used to guide ventilator settings remains to be determined.

## Supplementary Information

**Additional file 1.**

## Data Availability

The datasets used and/or analysed during the current study are available from the corresponding author on reasonable request.

## Abbreviations

PEEP: Positive end-expiratory pressure
ARDS: Acute respiratory distress syndrome
BMI: Body mass index
EIT: Electrical impedance tomography
ODCL: Alveolar overdistension and collapse
GI: Global inhomogeneity index
Cdyn: Dynamic respiratory system compliance
CTS: Cardiothoracic surgery
APACHE: Acute physiology and chronic health evaluation
EELI: End-expiratory lung impedance
OD: Alveolar overdistension
CL: Alveolar collapse
