# Supplementary material for: The global inhomogeneity index assessed by electrical impedance tomography overestimates PEEP requirement in patients with ARDS: an observational study
Source: BMC Anesthesiol. 2022 Aug 15;22:258. doi: 10.1186/s12871-022-01801-7 (PMC9377133; doi:10.1186/s12871-022-01801-7)
Supplement: Supplementary file 1 — Additional file 1. [file 12871_2022_1801_MOESM1_ESM.docx]

# The global inhomogeneity index assessed by electrical impedance tomography overestimates PEEP requirement in patients with ARDS: an observational study

**Supplementary Material**

*Journal:*

BMC Anesthesiology

*Authors:*

Serge J.H. Heines^1^, Sebastiaan A.M. de Jongh^1^, Ulrich Strauch^1^, Iwan C.C. van der Horst^1,2^, Marcel C.G. van de Poll^1,3,4^, Dennis C.J.J. Bergmans^1,4^

1. Department of Intensive Care Medicine, Maastricht University Medical Centre+, Maastricht, the Netherlands
2. Cardiovascular Research Institute Maastricht (CARIM), Maastricht University, Maastricht, The Netherlands
3. Department of Surgery, Maastricht University Medical Centre+, P. Debyelaan 25, 6229HX Maastricht, the Netherlands
4. School of Nutrition and Translational Research in Metabolism (NUTRIM), Maastricht University, Maastricht, the Netherlands

*Corresponding author:*

S. Heines, Department of Intensive Care, Maastricht University Medical Centre+, Maastricht, The Netherlands

P. Debyelaan 25, 6202 AZ Maastricht, The Netherlands, E-mail: s.heines@mumc.nl

**Supplementary Table 1** Optimal PEEP settings

| **PEEP based on** | **ARDS** | **Control** | **p-value** |
| --- | --- | --- | --- |
| best ODCL, cmH_2_O (SD) | 10.9 (2.5) | 9.6 (1.6) | 0.092 |
| lowest GI, cmH_2_O (SD) | 17.1 (3.9) | 14.2 (3.4) | 0.608 |
| GI <4% decrease, cmH_2_O (SD | 12.6 (4.0) | 9.8 (2.5) | 0.012* |
| best Cdyn, cmH_2_O (SD) | 10.3 (2.9) | 9.8 (2.5) | 0.864 |

Data are presented as means ± SD. ODCL, balance between alveolar overdistension and collapse; GI, global inhomogeneity index; GI <4% decrease, lowest PEEP with a decrease of the GI with less than 4 percent; Cdyn, dynamic respiratory system compliance; ARDS, acute respiratory distress syndrome. (*p<0.05)

**Supplementary Table 2** Differences between optimal PEEP based on ODCL, GI, GI,4% and Cdyn

|  | **ARDS** | **p-value** | **Correlation (r)** |  | **Control** | **p-value** | **Correlation (r)** |
| --- | --- | --- | --- | --- | --- | --- | --- |
| **ODCL - GI** | -6.2 (3.3) | <0.001* | 0.535 |  | -4.6 (3.8) | <0.001* | 0.030 |
| **ODCL - Cdyn** | 0.6 (2.1) | 0.161 | 0.711 |  | -0.1 (2.2) | 0.826 | 0.526 |
| **GI - Cdyn** | 6.8 (3.1) | <0.001* | 0.622 |  | 4.5 (4.4) | 0.001* | 0.110 |
| **ODCL – GI <4%** | -1.71 (2.7) | <0.001* | 0.751 |  | -0.12 (2.6) | 0.271 | 0.283 |
| **GI <4% - Cdyn** | 2.29 (3.2) | <0.001* | 0.601 |  | 0.24 (2.6) | 0.103 | 0.409 |

Data are presented as means ±SD. ODCL, balance between alveolar overdistension and collapse; GI, global inhomogeneity index; Cdyn, dynamic respiratory system compliance; GI <4%, lowest PEEP with a decrease of the GI with less than 4 percent; ARDS, acute respiratory distress syndrome; r, Pearson’s correlation coefficient (*p<0.05)
